# Exploring and Promoting Prosocial Vaccination: A Cross-Cultural Experiment on Vaccination of Health Care Personnel

**DOI:** 10.1155/2016/6870984

**Published:** 2016-09-20

**Authors:** Robert Böhm, Cornelia Betsch, Lars Korn, Cindy Holtmann

**Affiliations:** ^1^School of Business and Economics, RWTH Aachen University, Templergraben 64, 52062 Aachen, Germany; ^2^Center for Empirical Research in Economics and Behavioral Sciences (CEREB) and Department of Psychology, University of Erfurt, Nordhäuser Str. 63, 99089 Erfurt, Germany

## Abstract

Influenza vaccination for health care personnel (HCP) is recommended particularly because it indirectly protects patients from contracting the disease. Vaccinating can therefore be interpreted as a prosocial act. However, HCP vaccination rates are often far too low to prevent nosocomial infections. Effective interventions are needed to increase HCP's influenza vaccine uptake. Here we devise a novel tool to experimentally test interventions that aim at increasing prosocially motivated vaccine uptake under controlled conditions. We conducted a large-scale and cross-cultural experiment with participants from countries with either a collectivistic (South Korea) or an individualistic (USA) cultural background. Results showed that prosocially motivated vaccination was more likely in South Korea compared to the US, mediated by a greater perception of vaccination as a social act. However, changing the default of vaccination, such that participants had to opt out rather than to opt in, increased vaccine uptake in the US and therefore compensated for the lower level of prosocial vaccination. In sum, the present study provides both a novel method to investigate HCP influenza vaccination behavior and interventions to increase their vaccine uptake.

## 1. Introduction

The first goal of the WHO Global Action Plan for Influenza Vaccines [1, 2] is to increase seasonal influenza vaccine uptake. Health care personnel (HCP) play an important role in relation to this objective, as multiplier both on the front line of the vaccinating staff [3] and in the chain of nosocomial infections (i.e., hospital-acquired infections). Therefore, influenza vaccination is recommended for HCP in more than 40 countries, such as Germany, France, UK, the United States of America, or South Korea [1, 4, 5]. Influenza vaccines, like most vaccines, provide both direct protection to the vaccinating individual (individual benefit of vaccination) and indirect protection to non-vaccinated others (social benefit of vaccination) [6–10]. Hence, influenza vaccination for HCP is important because it reduces transmission of influenza to patients, who are often immunocompromised and therefore more severely threatened by influenza infections than healthy persons [11]. In fact, infections may be asymptomatic for healthy HCP and yet contagious and dangerous for patients. For instance, 23% of HCP in four UK clinics had serologic evidence of influenza virus infection during a single influenza season, but the majority reported only mild illness or subclinical infection [12]. Recent research suggests that HCP's influenza vaccination indeed prevents nosocomial infections and, as a result, reduces morbidity and mortality among patients [13].

Despite both the individual and the social benefit of seasonal influenza vaccination, immunization coverage among HCP is too low [14–16]. In Europe, uptake rates for seasonal influenza vaccine among HCP are below 32% [17]. The proportion of vaccinated HCP in the United States have risen from 40–50% to 60–70% due to intense promotion efforts and, in part, mandatory vaccination in some health care units [18]. Such low vaccination rates among HCP are dangerous because in hospitals the theoretical herd immunity threshold of 80% for influenza [19] may not be sufficient (by reducing the number of people who could get infected with the disease through vaccination, the basic reproductive rate of a pathogen (*R*_0_) could be reduced to a point where one primary case of infection produces less than one secondary case, resulting in the elimination of the disease [20, 21]; if the critical threshold of vaccination coverage in the population is reached, “herd immunity” is established [22]). In fact, epidemiologists recommend 100% vaccination coverage among HCP in order to reduce the infections by 60% [23, 24]. The large gap between the actual uptake and the uptake needed to provide optimal protection for patients underlines the importance of understanding and promoting prosocial vaccine uptake among HCP.

Previous research has shown that vaccinated HCP mention protection of their patients as the or one of the most important reasons for influenza vaccination [15, 25]. There is also ample other evidence that the prosocial aspect of vaccination influences the general decision in favor of or against a vaccination [7–9, 26]. In a simulated interactive vaccination game, for example, individuals vaccinated strategically and in line with their social preference: prosocial individuals, that is, those who also regard the outcomes of others in their decisions, were more likely to vaccinate than proself individuals, who focus solely on their own outcome [8]. Furthermore, scenario-based studies showed that vaccination intentions increased when other people's benefit was emphasized and the costs of vaccination were low [7, 27]. It therefore seems valuable to assess the interplay between the structure of the vaccination decision, the inclination for prosocial vaccination, and potential interventions to increase vaccine uptake among HCP.

Testing interventions to improve HCP's vaccine uptake in field experiments are effortful and costly. Therefore, it is reasonable to evaluate the effectiveness of potential interventions in lab or online experiments beforehand in order to save costs and time and to learn about the causal mechanisms under controlled conditions. Such artificial experiments, however, require valid and reliable measures that mirror the incentives of actual vaccination decisions. Behavioral games are an increasingly popular method from social and behavioral sciences that meet these requirements [28–30]. Behavioral games constitute simplified, well-defined models of real-life social decision situations. They capture the essential features of the situation of interest and exclude nonessential details. With this, they are able to illuminate the functional basis of motivational and behavioral processes that take place in the situation they aim to model. In turn, interventions that are effective in experiments using the behavioral game are likely to be successful in the actual field situation, too. Experimental game models of vaccination decisions have recently been introduced and successfully applied [8, 9, 31]. Such game models consider the associated costs of infection given non-vaccination and adverse events given vaccination, respectively. Similarly, they capture both the direct and the indirect effects of vaccination and therefore the social-interactive elements of vaccination.

In order to appropriately model the incentive structure of HCP influenza vaccination in a simplified way, we here devise a novel experimental game paradigm, the* HCP vaccination game*. In contrast to previous behavioral game models, the HCP vaccination game takes into account the externalities of HCP's vaccination decision on susceptible patients. As such, the incentives represent HCP's influenza vaccination as a purely prosocial action in the patients' interest. Certainly, HCP vaccination also entails personal benefits to the vaccinator. However, as argued above, the consequences to others (i.e., patients) are particularly important in the context of HCP influenza vaccination. In line with the general approach of behavioral game models to focus on essential incentives but to exclude non- or less-essential details, we therefore omit any personal benefits of HCP influenza vaccination. With this, we are able to investigate the predictors and potential promoters of prosocial vaccination in isolation (for a similar approach, see [25]). We conducted a large-scale cross-cultural experiment in order to investigate the predictors of vaccine uptake in the HCP vaccination game. In particular, we explored cultural differences in the level of prosocial vaccination between participants having an individualistic cultural background (i.e., participants from the USA) with participants having a collectivistic cultural background (i.e., participants from South Korea). Furthermore, we tested an intervention that can be particularly useful to improve vaccine uptake in the HCP context. Specifically, we manipulated the default option of the vaccination decision, that is, whether individuals need to opt-in or opt-out in order to receive a flu shot [32, 33]. The game structure of the HCP vaccination game as well as the manipulations and measures applied are explained in the next section.

## 2. Materials and Methods

### 2.1. The HCP Vaccination Game

In order to model the incentives of HCP's influenza vaccination decisions, we devise the following game structure: the game consists of 30 players, 10 HCP at a hospital ward and 20 patients. Players are endowed with health points representing their health status; HCP receive 10 health points each, whereas patients receive 5 health points. Participants are provided with information about a circulating disease. An infection with the disease entails particular risks for the patients in the care unit. Vaccination is possible for HCP only. A decision in favor of vaccination yields fixed costs of 2 points (e.g., effort needed to obtain vaccination, anxiety, or pain of pinprick). Moreover, after vaccination side effects may occur or not: in 1 of 3 cases, respectively, no side effects (no additional loss of health points), mild side effects (loss of 1 health point), or severe side effects will occur (loss of 2 health points). Thus, vaccination is costly: aggregating fixed costs and the possible costs due to side effects, vaccination leads to an expected loss of 3 health points for HCP. When not vaccinated, HCP will get infected. An infection does not result in any point loss for the HCP. Infected HCP can transmit the pathogen to their vulnerable patients who are not able to vaccinate themselves. When patients get infected with the disease, they suffer huge damage by losing 4 out of 5 health points. Whether patients will get infected depends on the number of vaccinated HCP employees, as a sufficient number of HCP have to get vaccinated in order to protect patients from contracting the disease. If this threshold in the HCP vaccination rate is reached, all patients are protected, whereas all patients will be infected when the HCP vaccination rate falls short of the threshold. The threshold was varied between subjects (see below).

This game presents a simplified version of the dilemma the HCP are facing in their professional environment regarding influenza vaccination. It especially emphasizes the prosocial aspect of HCP vaccination. As such, the HCP vaccination game constitutes a step-level volunteer's dilemma [34].

### 2.2. Experimental Factors

There were two versions of the HCP vaccination game, each requiring a different level of HCP vaccine uptake to protect patients effectively (7 versus 10 out of 10 HCP had to be vaccinated). Additionally, we considered participants' nationality as a proxy for the cultural background (individualistic versus collectivistic) and the default option of the vaccination decision (opt-in versus opt-out) as between-subjects factors in our experiment. Thus, the experiment implemented a 2 × 2 × 2 between-subjects quasi-experimental design. The following sections describe each of the experimental factors and present hypotheses regarding their impact on vaccine uptake in the HCP vaccination game.

#### 2.2.1. Herd Immunity Threshold

The HCP in the game are better off if they do not vaccinate, because vaccination is personally costly while they face no risk of point loss in case of an infection. In contrast, when the number of HCP who is willing to take the personal cost of vaccination is insufficient, all patients will lose considerable health points since the critical herd immunity threshold is not reached. Although providing the public good (i.e., reaching the vaccination threshold to protect patients) maximizes the collective welfare, contributions (i.e., vaccination) are costly and HCP have a strong incentive to free-ride (i.e., omitting vaccination); see Table 1. Therefore, non-vaccination is the selfish-rational strategy for HCP. When the level of vaccination among HCP exactly reaches the critical vaccination threshold, the collective optimum is constituted. In this experiment, we varied the collective optimum so that either seven or ten out of ten HCP needed to be vaccinated to protect the patients. A number of vaccinated HCP that exceeds the herd immunity threshold in the 7-of-10 condition (i.e., 8–10 HCP vaccinated) still maximizes social welfare compared to every number of vaccinated HCP that falls short of the critical vaccination threshold (see Table 1). This models the discussion around herd immunity thresholds in hospital settings, which are discussed to be between 75 and 100% [19, 23, 24]. Behavior in the game, that is, the decision for the rather selfish or prosocial option, should depend on the player's motivation to maximize the individual versus the social benefit. The next paragraph will go into more detail.

#### 2.2.2. Cultural Background

Vaccination in the devised HCP vaccination game is purely prosocial. Decisions in favor of vaccination prevent losses for patients and increase the social welfare without creating individual benefit for the vaccinated HCP. Further, vaccination causes costs solely for the vaccinated individuals. Only if the critical herd immunity threshold of vaccination coverage is reached patients can be eventually protected. With their decision to vaccinate, HCP are exclusively responsible for providing the public good. Moreover, contributing to the public good requires trust that other HCP will also vaccinate.

Research shows that individuals differ not only in their tendency to show prosocial behavior but also on a superordinate level such as culture [35, 36]. An influential construct to describe cross-cultural variability regarding prosocial behavior is collectivism versus individualism [37, 38], which refers to people of one group (culture) not the individual person. People from collectivistic cultures, for example, are assumed to be more influenced by social obligations and group norms compared to people from individualistic cultures [39, 40]. Thus, collectivists habitually focus on collective rather than on individual benefits while the reverse is true for individualists [41]. Because people with a collectivistic cultural background are more concerned with avoiding collective losses [42, 43] and are more likely to cooperate with in-group members [44], we hypothesized that prosocial vaccine uptake among subjects with a collectivistic cultural background is higher than among subjects with an individualistic cultural background. Furthermore, this higher vaccine uptake should be mediated by a greater acknowledgment of vaccination as a social act.

In order to test these hypotheses, we drew samples from South Korea and the US. South Koreans are assumed to be rather collectivistic, while US-Americans are assumed to be rather individualistic [45].

#### 2.2.3. Default Option of the Vaccination Decision

Defaults are preselected options that become effective if the decision-maker does not take any action to change them. In the context of vaccination decisions, non-vaccination is typically the default option and subjects have to opt-in by scheduling an appointment to receive the vaccination. Providing subjects with a previously scheduled vaccination appointment changes the default such that they have to become active in order to opt-out, that is, cancelling the appointment. It has been shown that vaccine uptake increases when vaccination rather than non-vaccination is the default option [9, 44]. Changing the default may be particularly effective when individuals do not have a strong preference in favor or against vaccination [46].

Given that in general people have some concern for others' welfare [47], changing the default from opt-in to opt-out is hypothesized to be particularly effective in increasing vaccination when prosocial concerns (and social norms elevating them) are less salient. This should rather be the case in individualistic cultures. In contrast, in collectivistic cultures prosocial vaccination is assumed to build the intrinsic default anyway (see above). Hence, we hypothesized that prosocial vaccine uptake is more strongly affected by changing the default of the vaccination decision among subjects from the US than among subjects from South Korea.

### 2.3. Participants

We conducted an online experiment via EFS survey; data from US participants was gathered with the crowdsourcing platform Microworkers; South Korean participants were recruited with an email distribution list provided by the Transgovernmental Enterprise for Pandemic Influenza (TEPIK). Two data collection intervals were needed to recruit participants in both subsamples. The moment of data collection varied in the South Korean and the US sample. Whereas in the US sample data was accessed in March 2015, the South Korean sample was accessed from May until July 2015.   *N* = 867 participants (*n* = 404 from the US and *n* = 463 from South Korea) completed the questionnaire, stated to have either US or South Korean nationality, answered questions that controlled for full encoding of the instructions correctly, and had a reasonable processing time between about 4 min and 30 min (M = 9 min and 15 s and SD = 4 min and 29 s, excluding the fastest and slowest 2.5% of the sample). The gender distribution was as follows: 45.6% female (*n* = 395; USA: 48.5% and South Korea: 43.0%) and 54.4% male (*n* = 472; USA: 51.5% and South Korea: 57%). The participants' mean age was 29.3 years (SD = 9.5), with participants from the US being slightly older (M = 32.4; SD = 11.6) than those from South Korea (M = 26.6; SD = 6.2). Approximately 32.4% of the US participants and 78.6% of the South Korean participants had a bachelor's or a higher educational degree.

### 2.4. Procedure

After the participants had provided informed consent, we asked for the participant's nationality in order to ensure that only people from the USA and South Korea entered the final data set. They were randomly assigned to one of the experimental conditions. The study consisted of a main task, that is, the HCP vaccination game as described above, and an additional short questionnaire to collect background factors. A progress bar indicated how much of the study was left at any time of the study.

Participants were asked to imagine that they belonged to a group of 10 HCP at a hospital ward with 20 patients. The participants were told to make all decisions from the perspective of a HCP. Participants were then provided with information about a fictitious circulating disease. They received all relevant information and values of potential point losses for HCP and patients. They learned that the risk for patients was contingent on HCP's vaccination decisions. This information was equal for all participants and varied only in the herd immunity threshold required to protect the patients (7 versus 10 vaccinated HCP out of 10).

Participants in the opt-in condition were provided with the opportunity to contact the occupational health service to make a personal appointment. If they wanted to vaccinate, they had to write an email to the occupational health service on the subsequent page of the study. An individual vaccination appointment with fixed date and time was suggested to participants in the opt-out condition. If they wanted to cancel the date, they too had to write an email to the occupational health service on the subsequent page.

Before participants made their decision to arrange or cancel the appointment, they were asked to express their beliefs about how many of the other 9 HCP will get vaccinated against the disease. After participants made their decisions, they received a summary of their individual vaccination decision and the resulting consequences of their choice (loss of health points or no loss of health points due to side effects).

### 2.5. Background Factors

In a postexperimental questionnaire, participants were asked to indicate their attitude towards vaccination on a seven-point scale (“In general, do you rather …”; 1 = “*lean away from vaccination*” to 7 = “*lean towards vaccination*”). Similarly, the belief of others' attitude towards vaccination was assessed with “Overall, my circle of friends is…” (1 = “*completely against vaccination*” to 7 = “*completely in favor of vaccination*”). Finally, an additional item was used to assess participants' belief that vaccination is a prosocial act (“*I am certain that I can protect others, when getting vaccinated*”; 1 = “*disagree*” to 7 = “*agree*”).

As a final step, participants' demographics were collected that have been found influential on health intentions, such as gender [48], level of education as an indicator of social status [49], and age [48]. Participants then had the opportunity to leave a short comment regarding the study. They were finally thanked, debriefed, and dismissed. It was stressed again that all information regarding the disease and the vaccination was fictitious.

## 3. Results and Discussion

### 3.1. Results

**Figure 1:**
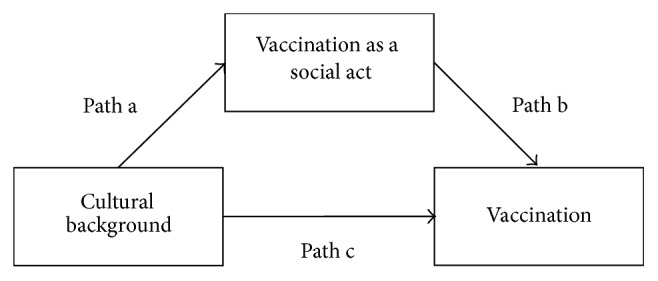
Tested mediation model.

The first hypothesis predicts greater vaccination coverage among subjects with a collectivistic cultural background compared to subjects with an individualistic cultural background. We compared the overall vaccination rate (coded: 0 = non-vaccination and 1 = vaccination) in South Korea (coded: 0) and the US (coded: 1) to test this hypothesis. Indeed, participants from South Korea were more likely to vaccinate than US participants;* B* = −1.11, SE = .23, and *p* < .001 (i.e., see Figure 1, path c). An odds ratio of 3.02 indicates that the odds of vaccination among South Koreans were about three times higher compared to US-Americans. Specifically, 94% of the South Korean participants and 83% of the US participants decided to vaccinate. People from a country with a collective orientation thus showed a higher level of prosocial vaccination than people with an individualistic cultural background. We further hypothesized that the effect of cultural differences on uptake should be mediated by the perception of vaccination as a social act (i.e., via path a and path b in Figure 1). We tested this mediation using a regression-based path-analytic framework [50]. In a first step, we found that participants from South Korea perceived vaccination as a social act to a larger degree than participants from the US (path a:* B* = −0.33, SE = .10, and *p* = .002). In a second step, greater perceptions of vaccination as a social act increased participants' likelihood to decide in favor of vaccination (while controlling for cultural background) (path b:* B* = 0.44, SE = .06, and *p* < .001). Lastly, the bootstrapped 95% confidence interval (bias-corrected and based on 5,000 iterations) of the indirect effect—cultural background affecting vaccine uptake via perceptions of vaccination as a social act—did not include zero and was therefore significant at a 5% level (path a*∗*b:* B* = −0.14, SE = .05, and 95% CI [−.25, −.06]). Hence, we found support for the hypothesized mediation pattern.

In order to work out the underlying predictors of vaccination behavior that may be specific to the cultural settings, we conducted generalized linear regression models with a logit link (logistic regression) separately for the US and South Korean subsample. Among US participants, using the structural manipulations default option, herd immunity threshold, and the respective interaction term as predictor variables (see Table 2, Model 1), we found that only the default option significantly affected vaccination behavior. As predicted, vaccination became more likely when it was the default option and participants had to actively opt-out compared to the condition where non-vaccination was the default option and participants had to actively opt-in. Taking participant's self-reported perceptions into account (attitude and beliefs; Model 2), we found that the participant's own attitude towards vaccination predicted vaccination behavior. Furthermore, the more positive the belief that others will vaccinate as well was, the more likely the participants' own vaccination was. Lastly, these effects remained stable when controlling for interindividual differences in age, gender, and education (Model 3).

Focusing on participants from South Korea, where vaccination likelihood was generally higher than among participants from the US (see above), the default manipulation did not significantly affect vaccinations, neither did the herd immunity threshold (see Table 3, Model 4). Adding the self-reported perceptions to the regression model (Model 5) yields that participants' own attitude towards vaccination and the belief that others will vaccinate increased the likelihood of vaccination (as among US participants; see above). Additionally, the perceived vaccination attitude of others increased vaccination. Furthermore, and in line with our hypothesis, a greater perception of vaccination as a social act increased the likelihood of vaccination. Lastly, these effects remained stable again when controlling for participants' age, gender, and education (Model 6).

### 3.2. Discussion

In sum, we found that people from different cultural backgrounds have different inclinations to vaccinate in a context where vaccination was (artificially made) purely prosocial. That is, participants from a collectivistic country (South Korea) were more willing to vaccinate prosocially than participants from an individualistic country (USA). This effect was mediated by the perception of vaccination being a prosocial act. The collectivistic orientation may also be related to the effect that the perceived attitude of relevant others impacts the decision to vaccinate. As such, the results provide evidence for different inclinations to get vaccinated based on basic psychological differences due to individuals' cultural background over and above structural and political differences between countries, for example, vaccination policies and mandates. Additionally, changing the default of vaccination appears to be an intervention apt to increase vaccine uptake in health care settings. This intervention proved successful, however, only in the more individualistic country (USA), where the general inclination to prosocial vaccination was lower. Previous research that showed the success of this intervention, too, has also been conducted in the Western societies (US and the Netherlands; [9, 31]). Among participants from a collectivistic country, however, the already higher proneness of prosocial vaccination did not further increase due to changing the default option. This corresponds with real vaccine uptake as influenza vaccination among HCP in South Korea is remarkably high with 82.3% [50]. Even if we assumed that the default intervention had no effect in South Korea due to a ceiling effect, we still may conclude that such interventions do have a culture-sensitive aspect [51], as in the present case culture determined differences in prosociality. Default manipulations in vaccination decisions may be particularly successful when there is a lack of prosocially motivated vaccination due to cultural or institutional conditions. Participants from both cultures were more willing to vaccinate when they believed that others would vaccinate too. This shows that trust in others' willingness to vaccinate is important, potentially especially in face of prosocial vaccination and when high uptake is required to protect others.

The game setting that was used in this study has some limitations. We chose parameters, such as the vaccination's lack of individual benefit for HCP or the massive negative effect for patients when the herd immunity threshold was not reached, to assess behavior under such conditions. While on the one hand this may compromise external validity, such exaggerations are, on the other hand, only possible in game settings and therefore important to assess behavior under specific or extreme conditions. In real-life settings, vaccination of HCP is not purely prosocial, but it also reduces the personal risk of being infected. Additionally, the risk of infection for patients is a linear rather than a step-level function of the vaccine uptake around them [20]. The fact that all patients became sick when the critical threshold was not reached may have contributed to the overall high vaccine uptake. Other variants of interactive vaccination games (such as the I-Vax game [8]) model disease risk as a linear function of vaccine uptake. Results of such studies show that decision-makers calibrate their decision to a selfish-rational optimum, adaptive to the vaccination risks and disease risks resulting from the uptake in the community. Future variants of the HCP vaccination game should therefore also include such an individual versus social benefit calculation in the game incentives. Moreover, they should allow for variations in the disease risk for patients. The rather extreme costs for patients could have suppressed potential effects of the threshold manipulation, which we found to not matter in participants' vaccine uptake. Nevertheless, the applied parameterization suggests that HCP will vaccinate when the potential damage for patients is high and when they feel that their own vaccination can protect the vulnerable patients.

The moral argument of patient protection is used very often in the discussion about mandatory vaccination for HCP [18]. It is often contrasted with low influenza vaccine effectiveness [52] and the fact that HCP are not the only source of infection in a hospital [53]. Doubts about whether herd immunity can be reached at all in such settings [23, 24] further question the legitimacy of demanding mandatory vaccination for HCP. Yet, studies that do show social benefit of high influenza vaccine uptake in hospital and nursery home settings may not be neglected [13, 54]. Thus, it seems necessary to identify strategies to increase HCP vaccine uptake that are effective yet not mandatory. The present research shows that there are several potential ways to increase influenza vaccine uptake among HCP, which are less paternalistic. First, changing the default from opt-in to opt-out can increase uptake, particularly in individualistic societies such as the US. This intervention can be interpreted as a strategy rooted in libertarian paternalism [55]: libertarian because individuals maintain their freedom of choice and paternalistic because the choice architecture is constructed in a way that fosters the desired choice. The second possibility of increasing HCP vaccine uptake is emphasizing the social benefit in hospital-bound vaccination campaigns. This conclusion is based on the finding that the belief of vaccination as a social act increased uptake. By emphasizing the social benefit, social preferences of HCP can be elicited, that is, increased, where they have been low previously [7, 56]. Yet, simply pointing to the social benefit may not be sufficient for a strong behavioral change. Other related studies show that priming empathy can increase hand hygiene behavior in hospitals [57]. As this can also be interpreted as prosocial behavior, future studies should test whether a combination of social benefit communication and priming of empathy can increase the effect of such interventions. They seem promising especially in communities or cultures that are rather individualistic than prosocially oriented, as the current study shows.

## 4. Conclusions

Increasing vaccine uptake in HCP is of utmost importance both to protect vulnerable patients in hospital settings and for increasing general influenza vaccine uptake [1, 2]. This study tests the determinants of vaccination behavior in a setting in which vaccination is assumed to be purely prosocial. The results show that person- and culture-bound prosociality and perceiving vaccination as a prosocial act explain vaccination behavior. This provides potential levers for vaccine advocacy. Strategies or campaigns that activate prosocial motives can increase prosocial vaccination [7, 27, 56]. Alternatively, nudges that change the default may be particularly successful when there is a lack of prosocially motivated vaccination due to cultural or institutional conditions. Experimental methods such as the HCP vaccination game provide helpful insights into the potential success of interventions and, in turn, may help to increase uptake by designing effective interventions.

## Figures and Tables

**Table 1:** Aggregated social welfare and payoffs for (non)vaccinated HCP and patients, depending on the number of vaccinated HCP in both versions of the HCP vaccination game.

HCP vaccinated	Payoff vaccinated HCP for game version A (B)	Payoff non-vaccinated HCP for game version A (B)	Payoff patients for game version A (B)	Social welfare for game version A (B)
0	n/a	10 (10)	1 (1)	120 (120)
1	7 (7)	10 (10)	1 (1)	117 (117)
2	7 (7)	10 (10)	1 (1)	114 (114)
3	7 (7)	10 (10)	1 (1)	111 (111)
4	7 (7)	10 (10)	1 (1)	108 (108)
5	7 (7)	10 (10)	1 (1)	105 (105)
6	7 (7)	10 (10)	1 (1)	102 (102)
7	7 (7)	10 (10)	5 (1)	**179 **(99)
8	7 (7)	10 (10)	5 (1)	176 (96)
9	7 (7)	10 (10)	5 (1)	173 (93)
10	7 (7)	n/a	5 (5)	170 (**170**)

*Notes*. Game version: A: critical vaccination threshold: 7 HCP; game version B: critical vaccination threshold: 10 HCP. Bold: social welfare optimum.

**Table 2:** Generalized linear regression models with a logit link, predicting vaccination behavior in the US subsample (*n* = 404).

Predictor	Model 1		Model 2		Model 3	
	*B*	SE	*B*	SE	*B*	SE
(Intercept)	.967^*∗∗∗*^	.226	−2.547^*∗∗∗*^	.668	−2.567^*∗∗*^	.866
Default option	1.146^*∗∗*^	.391	1.172^*∗∗*^	.430	1.178^*∗∗*^	.432
Herd immunity threshold	.312	.331	.097	.373	.086	.375
Default option × herd immunity threshold	.050	.589	.117	.640	.167	.645
Attitude of self			.417^*∗∗∗*^	.101	.399^*∗∗∗*^	.103
Belief attitude of others			−.108	.124	−.119	.126
Belief others vaccinated			.203^*∗∗*^	.074	.206^*∗∗*^	.077
Belief vaccination is a social act			.144	.099	.160	.101
Age					−.008	.014
Gender					−.061	.307
Education					.120	.102

*Notes*. Vaccination: 0 = non-vaccination and 1 = vaccination. Default option: 0 = opt-in and 1 = opt-out. Herd immunity threshold: 0 = 7 out of 10 and 1 = 10 out of 10. Gender: 0 = male and 1 = female. Education: 0 = below bachelor's degree and 1 = bachelor's degree or above. Significance levels: ^*∗*^ *p* < .05, ^*∗∗*^ *p* < .01, and ^*∗∗∗*^ *p* < .001.

**Table 3:** Generalized linear regression models with a logit link, predicting vaccination behavior in the South Korean subsample (*n* = 463).

Predictor	Model 4		Model 5		Model 6	
	*B*	SE	*B*	SE	*B*	SE
(Intercept)	2.438^*∗∗∗*^	.348	−7.426^*∗∗∗*^	1.416	−9.987^*∗∗∗*^	2.481
Default option	.404	.522	.278	.672	.279	0.702
Herd immunity threshold	.093	.491	−.613	.620	−.709	.636
Default option × herd immunity threshold	.264	.808	1.310	1.057	1.464	1.091
Attitude of self			.535^*∗∗∗*^	.152	.545^*∗∗∗*^	.152
Belief attitude of others			.631^*∗∗∗*^	.191	.614^*∗∗*^	.190
Belief others vaccinated			.481^*∗∗∗*^	.119	.481^*∗∗∗*^	.119
Belief vaccination is a social act			.320^*∗*^	.153	.336^*∗*^	.157
Age					.045	.057
Gender					.518	.522
Education					.049	.358

*Notes*. Vaccination: 0 = non-vaccination and 1 = vaccination. Default option: 0 = opt-in and 1 = opt-out. Herd immunity threshold: 0 = 7 out of 10 and 1 = 10 out of 10. Gender: 0 = male and 1 = female. Education: 0 = below bachelor's degree and 1 = bachelor's degree or above. Significance levels: ^*∗*^ *p* < .05, ^*∗∗*^ *p* < .01, and ^*∗∗∗*^ *p* < .001.
